# TGF-β and SHH Regulate Pluripotent Stem Cell Differentiation into Brain Microvascular Endothelial Cells in Generating an In Vitro Blood–Brain Barrier Model

**DOI:** 10.3390/bioengineering10101132

**Published:** 2023-09-27

**Authors:** Na Geum Lee, Mi-Hee Lim, Jongjin Park, In Cheul Jeung, Byungtae Hwang, Jangwook Lee, Jong-Gil Park, Mi-Young Son, Baek Soo Han, Sung-Jin Yoon, Seon-Jin Lee, Young-Jun Park, Jae Ho Kim, Nam-Kyung Lee, Sang Chul Lee, Jeong-Ki Min

**Affiliations:** 1 Biotherapeutics Translational Research Center, Korea Research Institute of Bioscience and Biotechnology, 125 Gwahak-ro, Yuseong-gu, Daejeon 34141, Republic of Korea; 2 Department of Biomolecular Science, KRIBB School of Bioscience, Korea University of Science and Technology, Daejeon 34141, Republic of Korea; 3 Department of Obstetrics and Gynecology, College of Medicine, The Catholic University of Korea, Jung-gu, Daejeon 34943, Republic of Korea; 4 Stem Cell Research Center, Korea Research Institute of Bioscience and Biotechnology, 125 Gwahak-ro, Yuseong-gu, Daejeon 34141, Republic of Korea; 5 Biodefense Research Center, Korea Research Institute of Bioscience and Biotechnology, 125 Gwahak-ro, Yuseong-gu, Daejeon 34141, Republic of Korea; 6 Environmental Disease Research Center, Korea Research Institute of Bioscience and Biotechnology, 125 Gwahak-ro, Yuseong-gu, Daejeon 34141, Republic of Korea; 7 Department of Physiology, Pusan National University Yangsan Hospital, Yangsan 50612, Republic of Korea; 8 Metabolic Regulation Research Center, Korea Research Institute of Bioscience and Biotechnology, 125 Gwahak-ro, Yuseong-gu, Daejeon 34141, Republic of Korea

**Keywords:** human pluripotent stem cells, brain microvascular endothelial cells, blood–brain barrier, differentiation, sonic hedgehog

## Abstract

Blood–brain barrier (BBB) models are important tools for studying CNS drug delivery, brain development, and brain disease. In vitro BBB models have been obtained from animals and immortalized cell lines; however, brain microvascular endothelial cells (BMECs) derived from them have several limitations. Furthermore, obtaining mature brain microvascular endothelial-like cells (BME-like cells) from human pluripotent stem cells (hPSCs) with desirable properties for establishing BBB models has been challenging. Here, we developed an efficient method for differentiating hPSCs into BMECs that are amenable to the development and application of human BBB models. The established conditions provided an environment similar to that occurring during BBB differentiation in the presence of the co-differentiating neural cell population by the modulation of TGF-β and SHH signaling. The developed BME-like cells showed well-organized tight junctions, appropriate expression of nutrient transporters, and polarized efflux transporter activity. In addition, BME-like cells responded to astrocytes, acquiring substantial barrier properties as measured by transendothelial electrical resistance. Moreover, the BME-like cells exhibited an immune quiescent property of BBB endothelial cells by decreasing the expression of adhesion molecules. Therefore, our novel cellular platform could be useful for drug screening and the development of brain-permeable pharmaceuticals.

## 1. Introduction

The blood–brain barrier (BBB) is a complex and selective system between the central nervous system (CNS) and the blood. It is composed of brain microvascular endothelial cells (BMECs) collaborating with perivascular cells, such as pericytes, astrocytes, and neurons, which form neurovascular units (NVUs) [1,2]. BMECs are distinguished from other endothelial cells in the expression of specialized tight junction (TJ) proteins and transporter systems for the import and export of molecules and low expression of leukocyte adhesion molecules (LAMs), limiting immune cell infiltration into the healthy brain [3,4].

Furthermore, the BBB has an important role in controlling molecular and cellular trafficking between the blood and neural tissue or its fluid spaces [5]. This barrier prevents the uptake of many toxic molecules and pathogens, and a disruption of the BBB leads to several neurodegenerative diseases [6]. Numerous therapeutic drugs for the brain have been developed to combat neurodegenerative diseases, but the delivery and penetration of drugs through the brain are low due to the BBB, posing difficulties for clinical applications and studies of the BBB system [7,8]. To overcome these limitations, the development of in vitro BBB models as a cellular platform for the discovery of therapeutic drugs, evaluation of drug toxicity, and innovative approaches for drug delivery across the BBB is warranted [8].

In vitro BBB models have been generated using primary BMECs (pBMECs) isolated from vertebrates and immortalized cell lines, as well as primary human brain microvascular endothelial cells (hBMECs). However, several problems limit the development and application of such BBB models, e.g., the inability of animal-derived cells to mimic the characteristics and functions of the human brain due to species differences, infrequent availability, loss of BMEC phenotype, and low barrier properties [9,10,11,12,13]. To surmount these hurdles, pluripotent stem cell (PSC) technologies have been highlighted as an attractive approach owing to their potential of differentiation into desired cell types and unlimited self-renewal [14].

Various methods to generate endothelial cells (ECs) possessing BBB properties from human PSCs (hPSCs) have been described thus far. ECs are differentiated from hPSCs, and purified cells are co-cultured with neural cells, such as astrocytes, pericytes, and neurons, to obtain BBB properties [15,16]. Furthermore, BMECs are obtained by a co-differentiation step of ECs with neural progenitor cells for the differentiation of ECs having BBB phenotype and functions [15]. Retinoic acid has been found to improve BBB phenotypes and enhance physical barrier properties such as transendothelial electrical resistance (TEER) values in hPSC-derived BMECs [5]. However, the differentiation system for hPSCs into BMECs with functional BBB properties is beset with limitations [14,17,18].

Meanwhile, the central nervous–vascular system communicates with multiple molecular signaling pathways during development [19,20]. The transforming growth factor-beta (TGF-β) signaling pathway is particularly crucial for embryonic development as it regulates pathophysiological processes of various organs [21]. Inhibition of the TGF-β signaling pathway accelerates the differentiation of neural- or endothelial-lineage cells, although the exact role of TGF-β signaling might vary depending on cell type and environment [22,23,24]. Therefore, we hypothesized that the regulation of the TGF-β signaling pathway could play key roles in enhancing the efficiency of hPSC differentiation into BME-like cells and acquiring the BBB phenotype by inducing co-differentiation into neural cells and ECs. Sonic hedgehog (SHH) signaling is involved in the maintenance of BBB integrity and immune quiescence of BMECs [25]. Accordingly, the inhibition of the TGF-β signaling pathway might activate SHH signaling and thereby enhance hPSC differentiation efficiency into BME-like cells and BBB properties.

Considering the above background, we aimed to develop a robust and facile differentiation method mimicking the brain microenvironment to produce functional ECs with BMEC phenotypes and properties from hPSCs, which will be useful for understanding human BBB physiology and for scaling up drug discovery for brain diseases.

## 2. Materials and Methods

### 2.1. Cell Culture and Medium Composition

hPSCs (human ES cells (hESCs; H9, WiCell Research Institute) and established human iPS cells (hiPSCs; UND-iPSCs, FAD-iPSCs, and SPD-iPSCs)) were maintained on γ-irradiated mouse embryonic fibroblasts (MEF, CEFObio, Seoul, Republic of Korea) in standard unconditioned medium: DMEM/F12 (Thermo Fisher, Carlsbad, CA, USA) containing 20% Knockout Serum Replacer (Thermo Fisher), 1X MEM nonessential amino acids (Thermo Fisher), 0.1 mM β-mercaptoethanol (Thermo Fisher), and human basic fibroblast growth factor (bFGF; 4 ng/mL for hESCs and 10 ng/mL for hiPSCs; R&D system). DMEM/F12 basal medium for stage 1 and stage 2 comprised DMEM/F12 (Thermo Fisher), 1% nonessential amino acids (Thermo Fisher), 5 mg/mL stem cell-grade bovine serum albumin (BSA; MP Biomedicals, Santa Ana, CA, USA), 10 µg/mL insulin (Sigma, St. Louis, MO, USA), 15 µg/mL holo-transferrin (Sigma), 450 µM 1-thioglycerol (Sigma), and 1X N2 supplement (100×) and 1X B27 supplement (50×) (Thermo Fisher). Basal medium for stage 3 consisted of human endothelial-serum-free medium (EC-SFM; Thermo Fisher) and 1% platelet-poor plasma-derived bovine serum (Sigma). Complete medium for stage 1 consisted of DMEM/F12 basal medium, 10 ng/mL Activin A (R&D system), 10 ng/mL BMP4 (R&D system), and 2 µM BIO (Sigma). Complete medium for stage 2 comprised DMEM/F12 basal medium, 20 ng/mL bFGF (Millipore, Burlington, MA, USA), and 10 µM SB431542 (Sigma). Complete medium for stage 3 consisted of EC-SFM basal medium, 20 ng/mL bFGF, 50 ng/mL vascular endothelial growth factor (VEGF) (R&D system), and 10 µM SB431542. hPSC-derived BME-like cells (stage 5) were cultured in endothelial cell growth medium (EGM-2; Lonza, Basel, Switzerland) with full supplements (EGM-2 bullet kit), 10% fetal bovine serum (FBS; Hyclone, Logan, UT, USA), 20 ng/mL bFGF, and 50 ng/mL VEGF. Primary human brain microvascular endothelial cells (pBMECs, ACBRI 376) were maintained according to the manufacturer’s instructions (Cell Systems).

### 2.2. Differentiation and Isolation of hPSC-Derived BME-like Cells

Before differentiation, cells were split on matrigel-coated plates in mTeSR medium (STEMCELL Technologies, Cambridge, MA, USA). After 2 days in mTeSR, the medium was switched to complete stage 1 medium. On day 3, the cells were switched to complete stage 2 medium, followed by 4 days in complete stage 3 medium. At the end of the differentiation period, cells were dissociated into single cells with accutase (Millipore), and dissociated cells were sorted into CD31-positive ECs by a magnetic assembly cell sorter (MACS) (Miltenyi Biotec, Cologne, Germany) using the CD31 microbead kit (Miltenyi Biotec). In brief, cell suspensions were passed through a 70 µm cell strainer (BD Biosciences, Franklin Lakes, NJ, USA) to remove cell aggregates, and cell counts were obtained. Up to 5 × 10^6^ cells were resuspended in 500 µL MACS buffer (1X PBS supplemented with 0.5% BSA and 2 mM EDTA) and incubated for 30 min at 4 °C with 25 µL FcR blocking reagent and CD31 microbeads (provided in the CD31 microbead kit). After the incubation, cells were washed with MACS buffer and then resuspended in 1 mL MACS buffer. Cell suspensions were loaded onto MS separation columns (Miltenyi Biotec). After the column was sufficiently washed with MACS buffer, CD31-positive cells were eluted in MACS buffer and centrifuged at 1500 rpm for 3 min at room temperature. The pellets were resuspended with EGM-2 with full supplements, 10% FBS, 20 ng/mL bFGF, and 50 ng/mL VEGF and then plated onto fibronectin-coated 35 mm dishes for culture.

### 2.3. Flow Cytometry Analysis

For fluorescence-activated cell sorting (FACS), cells were washed with PBS and dissociated with accutase (Millipore). Harvested cells were washed with FACS buffer (PBS supplemented with 2% FBS). Cells were fixed with fixation solution (BD Biosciences) for 20 min at 4 °C and then washed using FACS buffer. Cells were resuspended in 100 µL FACS buffer and incubated with the corresponding antibodies for 30 min at 4 °C in the dark. After the incubation, cells were washed with FACS buffer and labeled with fluorochrome-conjugated secondary antibodies (Thermo Fisher) for 30 min at 4 °C in the dark only if using unconjugated primary antibody. After incubation, cells were washed twice with FACS buffer and resuspended in a total volume of 400 µL before analysis. Cells were analyzed by using BD FACSCalibur. Data analyses were performed using WinMDI2.9 software (BD Biosciences). The antibodies used for flow cytometry analysis are listed in Supplementary Table S1.

### 2.4. Western Blot Analysis

Cells were washed once with PBS and lysed with lysis buffer (50 mM Tris–HCl, pH 7.6, 150 mM NaCl, 1 mM ethylene diamine tetraacetic acid, 0.5% sodium deoxycholate, 1% Triton X-100, 50 mM β-glycerophosphate, 50 mM NaF, 1 mM Na_3_VO_4_, 1X protease inhibitor). Proteins were separated by sodium dodecyl sulphate–polyacrylamide gel electrophoresis and subsequently transferred to Immobilon-P membranes (Millipore, IPVH00010). Blots were developed using the Amersham ECL western blotting detection reagent (GE Healthcare, Uppsala, Sweden, RPN2106). β-Actin was used as a control. The primary antibodies used for western blot analysis are listed in Supplementary Table S1.

### 2.5. RNA Isolation and RT-PCR Analysis

The RNA isolation and RT-PCR analysis was performed as described [26]. Cells were washed with PBS and RNA extraction was performed using Trizol (Thermo Fisher) according to the manufacturer’s instructions. For cDNA synthesis, 2 µg of total RNA was reverse-transcribed using oligo(dT)18 and M-MLV reverse transcriptase (Promega Korea, Seoul, Republic of Korea), according to the manufacturer’s instructions. PCR amplification of target genes was performed using the GoTaq Green Master Mix (Promega), and qPCR was performed using 2X Real-time PCR Smart mix (Solgent, Daejeon, Republic of Korea). The PCR conditions were as follows: initial denaturation at 95 °C for 5 min; 30 cycles of denaturation at 95 °C for 30 s, annealing at 56–58 °C for 30 s, and extension at 72 °C for 30 s; and a final 10 min extension step at 72 °C. The primers used for the RT-PCR experiments are listed in Supplementary Table S2.

### 2.6. RNA-Sequence Analysis

After RNA isolation, as described in “Section 2.5”, the Illumina Truseq Stranded mRNA Sample Preparation kit protocol (Illumina, San Diego, CA, USA) was followed, using 500 ng RNA per sample as the starting material. Libraries were checked for quality and quantified using Bioanalyzer 2100 (Agilent, Santa Clara, CA, USA), before being sequenced in barcoded pools of 13 samples on the Illumina HiSeq 2500 instrument (100 base paired-end sequencing). After sequencing, the reads were aligned using TopHat v2.0.11 software against the reference genome (UCSC hg19 iGenome). Default options were used, except the fr-firststrand option for library type. Then, the transcript assembly step was processed with the mapped read files by Cufflinks package v2.2.1. Abundance per transcript and gene were estimated and normalized as fragments per kilobase of exon per million fragments mapped (FPKM) value based on the pre-existing gene model (hg19.iGenome.UCSC.genes). Differentially expressed genes were identified by the Cuffdiff module with a false discovery rate of 5%. Plot images (scatter/density/box/volcano) were generated by cummerbund v2.8.0 and R version 2.15.1. Data are deposited in GEO (GEO ID: GSE148534).

### 2.7. Immunocytochemistry and Fluorescence Microscopy

Cells were washed with PBS and fixed using 3.7% formaldehyde (Sigma) for 10 min at room temperature. For intracellular staining, cells were permeabilized with 0.2% Triton X-100 (Sigma) for 10 min at room temperature. The cells were then blocked with 1% BSA (GenDEPOT) in PBS for 1 h at room temperature and then incubated with the indicated primary antibody for 2 h, followed by Alexa fluorochrome-conjugated secondary antibodies (Thermo Fisher) for 1 h. 4′,6-Diamidino-2-phenylindole (DAPI; Sigma) staining was performed for nuclear visualization. Fluorescence image acquisition was performed using florescence microscopy, and data analysis was performed using Metamorph 7.1 software (Molecular Devices, San Jose, CA, USA). The antibodies used for the immunocytochemistry are listed in Supplementary Table S1.

### 2.8. Acetylated LDL Uptake Assay and Vascular Tube-like Structure Formation Assay

For acetylated LDL uptake, pBMECs (Cell Systems) or hPSC-derived BME-like cells were incubated with 5 µg/mL acetylated LDL labeled with the fluorescent probe 1,1′-dioctadecyl-3,3,3′,3′-tetramethyl-indocarbocyanine perchlorate (DiI-Ac-LDL; Thermo Fisher) for 12 h at 37 °C, washed with PBS, and visualized immediately with a fluorescence microscope. Data were analyzed using Metamorph 7.1 software. For vascular tube-like structure formation, 24-well plates were coated with 250 µL of growth factor-reduced (GFR) matrigel (Corning, New York, NY, USA) for 30 min at 37 °C. Next, pBMECs or hPSC-derived BME-like cells were seeded (1.2 × 10^5^ cells) into each matrigel-coated well in EBM-2 basal medium (Lonza) containing 1% FBS. After 16–24 h, the formation of tube-like structures was imaged under bright-field microscopy.

### 2.9. Cell Proliferation Analysis

pBMECs and BME-like cells (passages 1–10) were seeded in fibronectin-coated 24-well plates at 5 × 10^4^ cells per well. After 2 days, the cells were harvested with trypsin/EDTA (Gibco), and the number of cells was quantified by direct cell counting.

### 2.10. Monocyte Adhesion Assay

hPSC-derived BME-like cells and control HUVECs were seeded on fibronectin-coated 6-well plates at a density of 3.5 × 10^5^ cells/well in 10% FBS EGM-2 supplemented with 50 ng/mL VEGF and 20 ng/mL bFGF. The next day, these cells were starved in 1% M199 for 6 h and treated with 10 ng/mL tumor necrosis factor-alpha (TNF-α; R&D system) or interleukin 1-beta (IL-1β; R&D system) for 4 h. After 4 h, human monocyte THP-1 cells labeled with 0.5 µM calcein-AM (Thermo Fisher) in 1% RPMI (Thermo Fisher) for 30 min were added (1 × 10^6^ cells/100 µL) to the confluent hPSC-derived BME-like cells and HUVEC monolayers and incubated for 15 min. Thereafter, the cells in the wells were washed three times with PBS and fixed using 4% formaldehyde, and the adherent cells were observed under fluorescence microscopy.

### 2.11. Efflux Transport Assay

The functionalities of P-glycoprotein (P-gp), breast cancer resistance protein (BCRP), and multi-drug resistance protein (MRP) were assessed using mitoxantrone (Sigma), a fluorescent substrate for efflux transporters. pBMECs or hPSC-derived BME-like cells were seeded in 12-well plates at a density of 2 × 10^5^ cells per well and cultured in EGM-2 containing 10% FBS for 24 h. The cells were pre-incubated in EBM2 medium with or without 10 µM mitoxantrone for 30 min and then washed three times with cold PBS. After the cellular uptake of mitoxantrone, the cells were cultured for 12 h at 37 °C in EGM-2 containing 10% FBS with or without 25 µM MK571 (Sigma), 50 µM verapamil (Sigma), or 0.5 µM Ko143 (Sigma). At the end of the incubation, cells were washed three times with cold PBS and dissociated using accutase. Intracellular fluorescence intensities were measured using BD FACSCalibur. To assess apical to basolateral transport, astrocytes were seeded into the bottom of 24-well plates before pBMECs or hPSC-derived BME-like cell seeding. pBMECs or hPSC-derived BME-like cells were labeled with or without 0.5 µM calcein-AM for 30 min at 37 °C. The cells were washed twice with PBS, seeded onto fibronectin-coated 0.4 µm transwell inserts (Corning), and then co-cultured with astrocytes in 10% FBS containing EGM-2 with or without inhibitor (MK571) for 12 h at 37 °C. The transport of calcein-AM from pBMECs or hPSC-derived BME-like cells to astrocytes was measured using BD FACSCalibur. Data analyses were performed using WinMDI2.9 software (BD Biosciences).

### 2.12. Measurement of TEER and Permeability Assay

hPSC-derived BME-like cells were seeded on fibronectin-coated transwell inserts (0.4 µm 12-well transwell insert, Corning) in 10% FBS EGM-2 supplemented with 50 ng/mL VEGF and 20 ng/mL bFGF and then co-cultured with astrocytes and human embryonic kidney 293 (HEK293) cells for 12–72 h. Measurement of TEER values was according to the Millicell ERS-2 (Millipore) instructions.

To identify the permeability of FITC-dextran, hPSC-derived BME-like cells were seeded as indicated above. The next day, co-culture with astrocytes and HEK293 cells for 48 h was carried out. After 2 days, hPSC-derived BME-like cell culture medium was changed to EBM-2 containing 100 µg/mL FITC-dextran (70kDa) (Sigma) to measure the fluorescence intensity of FITC-dextran in the bottom medium. Fluorescence intensity was measured using a plate reader (485 nm excitation and 530 nm emission). Triplicate filters were used for TEER and permeability studies.

### 2.13. Quantification and Statistical Analysis

Statistical analyses were performed with Student’s two-tailed *t* test. The data are shown as means ± the standard error of the means. *p* values of <0.1 were considered to represent statistically significant differences.

## 3. Results

### 3.1. Differentiation of hPSCs into BME-like Cells

**Figure 1:**
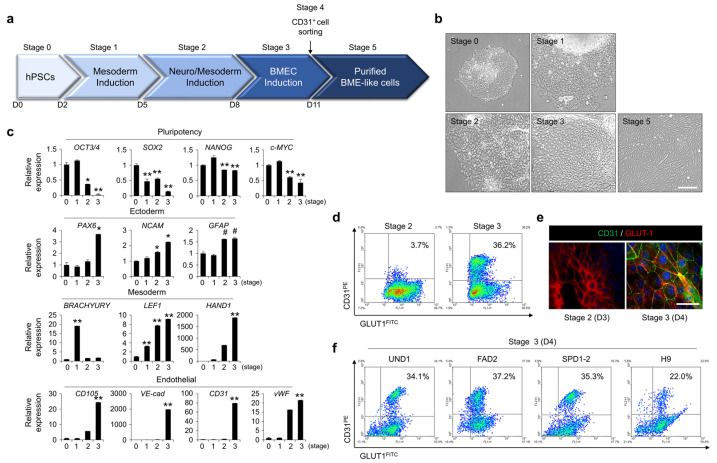
Differentiation of hPSCs into brain microvascular endothelial cells (BMECs). (**a**) Schematic detailing the five stages of differentiation and the accompanying growth factors, inhibitors, and basal medium needed for each stage. The approximate days required for each stage are indicated, and the detailed differentiation protocol is described in the ‘Materials and Methods’ section. (**b**) Morphological changes at stage of differentiation in hPSCs were observed under bright-field microscopy. Scale bar = 50 µm. (**c**) Gene expression for pluripotent stem cell markers (OCT3/4, SOX2, NANOG, and c-MYC), ectoderm markers (PAX6, NCAM1, and GFAP), and mesoderm markers (BRACHYURY, LEF1, and HAND1) was detected by real-time PCR at indicated stages of differentiation of hPSCs. The glyceraldehyde 3-phosphate dehydrogenase gene (GAPDH) was used as an internal control. # *p* < 0.1, * *p* < 0.05, ** *p* < 0.01 vs. stage 0. Data are represented as mean ± SEM. (**d**,**e**) The expression of CD31 and GLUT1 was assessed by FACS analysis (**d**) and immunocytochemistry (**e**) at indicated stages of differentiation of hPSCs. Scale bar = 200 µm. (**f**) hPSCs were differentiated from stage 0 to stage 3, and the expression of CD31 and GLUT1 was assessed by FACS analysis at stage 3. hPSC type and percentage of CD31+GLUT1+ subpopulations are indicated.

To obtain BME-like cells, hPSCs were differentiated using a stepwise differentiation protocol: stage 1 for definitive mesoderm, stage 2 for neuromesoderm, and stage 3 for mature BME-like cells. Finally, a pure CD31+ population was purified by magnetic assembly cell sorting (MACS) (stage 4) using CD31 microbeads. Sorted CD31+ cells were cultured in EC medium to expand the purified BME-like cell population (Figure 1a). Next, we identified the morphologies of differentiating cells at each stage and the expression of stage-specific markers (Figure 1b,c and Figure S1a). During these steps toward differentiation, while pluripotency-related markers (OCT3/4, SOX2, NANOG, and c-MYC) were downregulated after the start of the differentiation process, mesoderm markers such as BRACHYURY, LEF1, and HAND1 were rapidly upregulated (Figure 1c and Figure S1a). In parallel, the expression of several neuroectoderm markers, such as PAX6, NCAM, and glial fibrillary acidic protein (GFAP) (Figure 1c and Figure S1a), as well as EC-specific markers, including CD105, VE-cadherin (VE-cad), CD31, and von Willebrand factor (vWF), was significantly augmented during differentiation into mature BME-like cells (Figure 1c and Figure S1a).

Wnt signaling pathway is associated with mesoderm lineage differentiation and promotes BBB specification of ECs [15,27,28,29,30,31]. To identify whether activation of wnt signaling pathway regulates mesoderm-lineage induction, hPSCs were cultured in multiple combinations of components: Activin A (A), BMP4 (B), a glycogen synthase kinase 3β inhibitor, BIO, and neural cell-lineage supplements 1X N2 and B27 (N2B27). As shown in Figure S1b, when apply of BIO at stage 1, the expression of mesoderm-related genes such as BRACHYURY, LEF1, and HAND1, rather than ectoderm-related genes (PAX6 and SOX1), was significantly upregulated compared with untreated group of BIO.

To determine whether mature BME-like cells express the brain endothelial-specific glucose transporter GLUT1 [14,32,33], we analyzed the expression level of GLUT1, and, simultaneously, that of CD31 in differentiating cells at stage 3 by FACS and immunocytochemistry analysis. As shown in Figure 1d,e, the percentage of the CD31+GLUT1+ population was gradually increased during differentiation, and GLUT1 was co-expressed in the CD31-expressing population at stage 3. Moreover, other hPSCs represented similar percentages of the CD31+GLUT1+ population at the final differentiation stage by FACS analysis in the same manner (about 22–37%) (Figure 1f).

### 3.2. Similarity of BME-like Cells Derived from hPSCs with Primary BMECs

**Figure 2:**
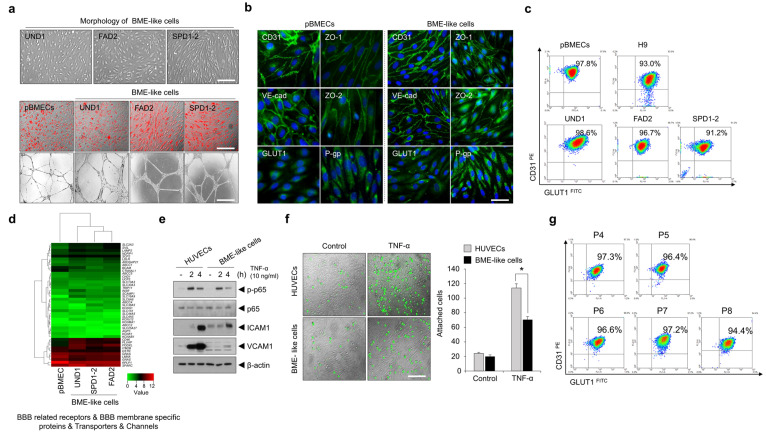
Characterization of hPSC-derived BME-like cells. hPSCs were differentiated into BME-like cells according to a differentiation protocol, and purified BME-like cells derived from hPSCs were characterized. (**a**) The morphology of hPSC-derived BME-like cells was observed under bright-field microscopy. Scale bar = 200 µm. Ac-LDL uptake and tube formation. Upper panel, pBMECs and hPSC-derived BME-like cells took up DiI-labeled Ac-LDL (red fluorescence), and then overlapping images of bright-field microscopy with fluorescent channels yielded fluorescent microscopy. Scale bar = 200 µm. Lower panel, pBMECs and hPSC-derived BME-like cells were seeded on GFR matrigel-coated plates and incubated for 16–24 h. Images of capillary-like tube formation were obtained under bright-field microscopy. Scale bar = 100 µm. (**b**) The expression of BMEC-related markers (CD31, VE-cad, GLUT1, ZO-1, ZO-2, and P-gp) was observed by immunocytochemistry. Scale bar = 50 µm. (**c**) The expression of CD31 and GLUT1 in pBMECs and hPSC-derived BME-like cells was assessed by FACS analysis. The percentage of the CD31+GLUT1+ subpopulation is indicated. (**d**) Heatmap of genes involved in the BBB related receptors, BBB membrane-specific proteins, transporters, and channels, demonstrating that hPSC-derived BME-like cells were similar to pBMECs. (**e**) HUVECs and hPSC-derived BME-like cells were treated with or without 10 ng/mL TNF-α for the indicated times. Then, the expression levels of p-p65, ICAM1, and VCAM1 were determined by western blot analysis. β-Actin was used as the loading control. (**f**) HUVECs and hPSC-derived BME-like cells were treated with 10 ng/mL TNF-α for 4 h. The cells were co-cultured with 0.5 µM of calcein-AM-labeled THP-1 human monocytes for 15 min. Left panel, overlapping of bright-field images with green fluorescence images. Right panel, bar graph for the number of THP-1 attached to HUVECs or hPSC-derived BME-like cells. Statistical significance is denoted by * *p* < 0.05 vs. HUVECs. Data are represented as mean ± SEM. Scale bar = 200 µm. (**g**) The expression of CD31 and GLUT1 in BME-like cells (passage 4–8) was assessed by FACS analysis even after continuous culture. The percentage of the CD31+GLUT1+ subpopulation is indicated.

To compare the whole-gene expression pattern of BME-like cells differentiated from hPSCs with pBMECs, RNA-sequence analyses were performed in hPSCs, BME-like cells, and pBMECs. A heatmap displaying hierarchical clustering results showed that hPSC-derived BME-like cells were similar to pBMECs (Figure S2a). Similarly, comparison of expressed genes in the purified BME-like cells and pBMECs indicated a high degree of similarity, with a correlation coefficient of over 0.900 (Figure S2b). Next, we evaluated BBB phenotype and functions to identify the similarity of BME-like cells with pBMECs. As shown in Figure 2a, BME-like cells presented typical endothelial morphology and capillary-like tube formation and could efficiently take up Ac-LDL. In addition, we observed that an EC-specific protein (CD31), TJ proteins (claudin5, occludin, ZO-1, and ZO-2), adhesion molecule (VE-cad), and a transporter (P-gp) were expressed at similar or higher levels compared with pBMECs (Figure 2b and Figure S2c,d). Furthermore, we confirmed that the expression of CD31 and GLUT1 in purified BME-like cells remained at 90–98%, similar to that in pBMECs, even after continuous culture (Figure 2c). Unlike conventional ECs, brain ECs have specific transporter systems and channels to transport several nutrients, ions, and molecules between the bloodstream and the brain [2,3]. These genes also show similar levels between BME-like cells and pBMECs (Figure 2d and Figure S2e–i).

CNS ECs surrounding parenchymal cells have low level of immune surveillance, which results in a paucity of entry of immune cells during BBB development and homeostasis [34]. In particular, the expression of leukocyte adhesion molecules (LAMs) in CNS ECs is extremely low compared with general ECs, even though expression of these molecules is upregulated under neuroinflammatory diseases [4,34]. Based on this background, to verify immune quiescence in BME-like cells derived from PSCs, HUVECs and BME-like cells were treated with inflammatory cytokines—10 ng/mL TNF-α or 10 ng/mL IL1-β—followed by the determination of the phosphorylation of p65, a component of NF-κB, and the expression of ICAM1 and VCAM1 by western blotting. Compared to HUVECs, BME-like cells exhibited lower expression levels of ICAM1 and VCAM1 and lower p65 phosphorylation levels (Figure 2e and Figure S2j). In addition, we observed that the attachment of monocytes (THP-1 cells) to BME-like cells was lower in response to TNF-α than in those of the control HUVECs (Figure 2f). Although several protocols to produce BME-like cells-derived from PSCs are available, the purified BME-like cells from these protocols were few and limited in functionality and/or longevity to study the BBB model. Notably, we identified that BME-like cells represented similar expression pattern of CD31 and GLUT1 despite passaging, which demonstrated that BME-like cells differentiated from our system maintained character of brain microvascular endothelial cells for a long time (Figure 2g). Together, all these results showed that the BME-like cells from our differentiation method may have functionally mature BMEC features exceeding those of previously reported BME-like cells. 

### 3.3. Assessment of BBB Properties of BME-like Cells Derived from hPSCs

**Figure 3:**
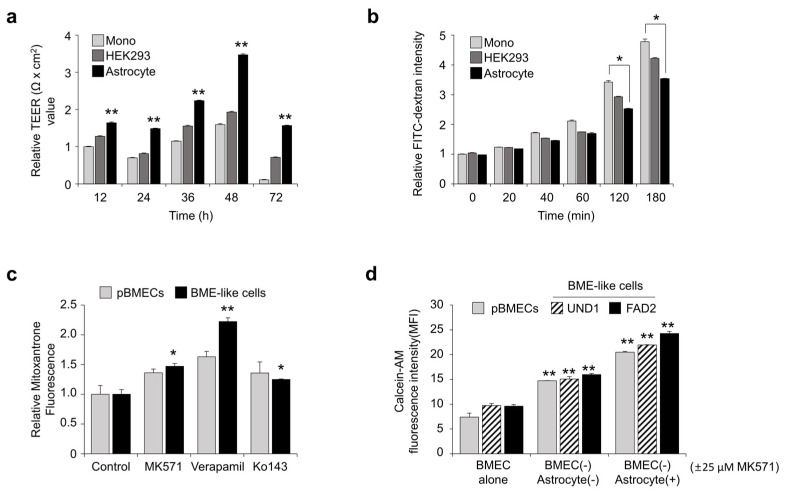
BBB functional properties of hPSC-derived BME-like cells. (**a**) TEER measured on hPSC-derived BME-like cells co-cultured with astrocytes or HEK293 cells under indirect-contact co-culture using transwell. Triplicate filters were used to calculate TEER values. Statistical significance is denoted by ** *p* < 0.01 vs. monoculture. Data are represented as mean ± SEM. (**b**) Permeability of FITC-dextran of hPSC-derived BME-like cells when co-cultured with astrocytes or HEK293 cells. Statistical significance is denoted by * *p* < 0.05 vs. monoculture. Data are represented as mean ± SEM. (**c**) pBMECs or hPSC-derived BME- like cells were taken up with mitoxantrone and then cultured with or without 25 µM MK571, 50 µM verapamil, or 0.5 µM Ko143 for 12 h. The intensity of intracellular fluorescence was measured by FACS analysis. Quantification from three independent assays is shown in the graphs. Statistical significance is denoted by * *p* < 0.05, ** *p* < 0.01 vs. control. Data are represented as mean ± SEM. (**d**) hPSC-derived BME-like cells and pBMEC were taken up with 0.5 µM of calcein-AM for 30 min, and then co-cultured with astrocyte with or without 25 µM of MK571. After 12 h, transport of calcein-AM from pBMECs or hPSC-derived BME-like cells (apical) to astrocytes (basolateral) was identified by measuring intensity of calcein-AM in astrocyte using flow cytometry. ** *p* < 0.01 vs. control. Data are represented as mean ± SEM.

Neural cells such as astrocytes, pericytes, and neurons, which enhance BBB properties and BMEC functions, particularly affect TEER and permeability [35]. To determine whether BBB properties of BME-like cells are enhanced when co-cultured with neural cells, we evaluated the TEER values and dextran permeability by co-culturing BME-like cells with non-neural HEK293 cells or astrocytes in comparison with monoculture. hPSC-derived BME-like cells were seeded onto fibronectin coated transwell and co-cultured with astrocyte, HEK293 cells, or nothing (i.e., monoculture). Co-culturing of BME-like cells with astrocytes significantly increased the TEER value (>twofold change) compared to mono-cultured BME-like cells or those co-cultured with HEK293 cells (Figure 3a). Moreover, FITC-dextran permeability of hPSC-derived BME-like cells was significantly decreased in a co-culture with astrocytes (Figure 3b), indicating that BBB properties of BME-like cells are characterized by increased BBB properties when co-cultured with neural cells.

Next, to investigate whether the efflux transporters such as P-gp, BCRP, and MRP are active, hPSC-derived BME-like cells were incubated with mitoxantrone, and intracellular fluorescence accumulation was measured in the presence of MK571, verapamil, and Ko 143, which are inhibitors of ABCC-MRP1, P-gp, and BCRP, respectively. Upon inhibiting pBMECs and BME-like cells with these inhibitors, accumulation of mitoxantrone in BME-like cells was increased to similar levels as in pBMECs (Figure 3c). Furthermore, to identify the efflux function, we determined the directional transport assay with BME-like cells (apical)-astrocytes (basolateral), representing brain-to-blood transit [15,36]. As shown in Figure 3d, similar to pBMECs, when hPSC-derived BME-like cells that had taken up calcein-AM were co-cultured with astrocytes in the presence or absence of MK571, an inhibitor of the MRP family, a higher accumulation of calcein-AM in astrocytes was found compared with the control, suggesting that the MRP transporter in BME-like cells has efflux activity and also increases efflux function when co-cultured with astrocytes. Taken together, these results indicate that the BME-like cells presented functional hBMEC properties and acted as a pertinent platform for studying the transport of therapeutic drugs for neural diseases.

### 3.4. Inhibition of TGF-β Signaling Is Involved in BBB Specification during BMEC Differentiation

**Figure 4:**
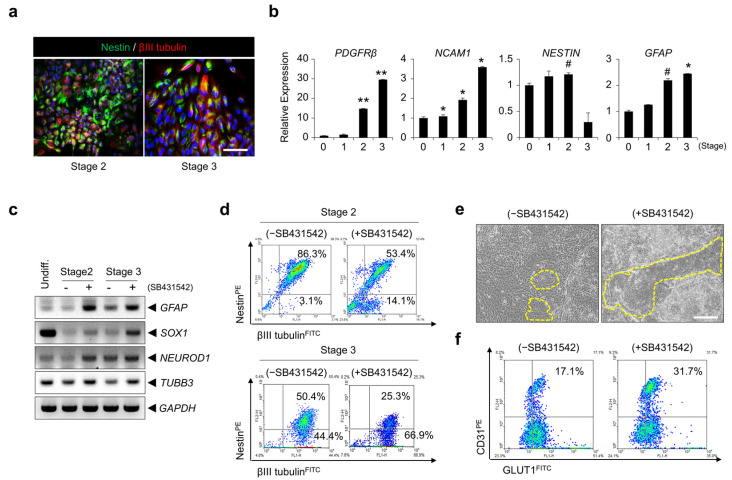
Inhibition of TGF-β signaling is involved in BBB specification during the differentiation of BME-like cells. (**a**) Representative fluorescence images of nestin (green) and βIII tubulin (red) during differentiation into BME-like cells from hPSCs at stage 2 and stage 3. Scale bar = 100 µm. (**b**) Gene expression levels of neural lineage-related markers (PDGFRβ: pericyte; NCAM1: neuron; NESTIN: neural progenitor; and GFAP: astrocyte) were analyzed by RT-PCR during differentiation into BME-like cells from hPSCs. # *p* < 0.1, * *p* < 0.05, ** *p* < 0.01 vs. stage 0. Data are represented as mean ± SEM. (**c**) Gene expression of neural lineage markers (GFAP, SOX1, NEUROD1, and TUBB3) were determined by RT-PCR during differentiation into BME-like cells from hPSCs in control (-SB431542) and 10 µM SB431542-treated (+SB431542), differentiating cultures at stage 2 and stage 3. (**d**) Comparison of nestin and βIII tubulin expression was assessed by FACS analysis during differentiation into BME-like cells from hPSCs in control (-SB431542) and 10 µM SB431542-treated (+SB431542), differentiating cultures at stage 2 and stage 3. (**e**,**f**) Endothelial cell-like morphology indicated in yellow lines was observed under bright-field microscopy (**e**), and the expression of CD31 and GLUT1 was determined by FACS analysis (**f**) during differentiation into BME-like cells from hPSCs in differentiating cultures with or without 10 µM SB431542 at stage 3. Scale bar = 200 µm.

Following culture in mesoderm induction medium, hPSCs were differentiated in neuromesoderm induction medium and BME-like cell induction medium with a TGF-β signaling pathway inhibitor, SB431542, for 3 days and 4 days, respectively. We identified that cells expressing nestin, a neural progenitor marker, were highly augmented at stage 2 and at stage 3, differentiating cells expressing βIII tubulin+, a neuron marker, were slightly increased (Figure 4a). Consistent with this, the expression of genes related to the neural lineage, such as PDGFR-β (pericyte), NCAM1 (neuron), NESTIN (neural progenitor), and GFAP (astrocyte), increased during these stages (Figure 4b). To determine if the inhibition of TGF-β signaling contributes to the differentiation of a neural population, we identified neural cell-related marker expression during differentiation with or without the addition of SB431542. Gene expression levels of GFAP, SOX1, NEUROD1, and TUBB3 increased with SB431542 treatment compared with the control (Figure 4c). Furthermore, the nestin−βIII tubulin+ population was higher with SB431542 treatment than the control at stage 2 and stage 3 by FACS analysis (Figure 4d). Next, we investigated whether inhibition of the TGF-β signaling pathway enhances the differentiation potential into brain ECs. As shown in Figure 4e, we observed that endothelial sheets (yellow line) were significantly increased. In addition, the CD31+ population expressing GLUT1 was significantly higher in cells treated with SB431542 (1.5- to 2-fold) compared with the control (Figure 4f and Figure S3). These results suggest that inhibition of the TGF-β signaling pathway may enhance the efficiency of hPSC differentiation into BME-like cells, at least in part, by conferring the potential of differentiation into neural cells, which is essential for BBB development.

### 3.5. TGF-β Signaling-Mediated SHH Expression Is Important for hPSC Differentiation into BME-like Cells and the Acquisition of BBB Properties

**Figure 5:**
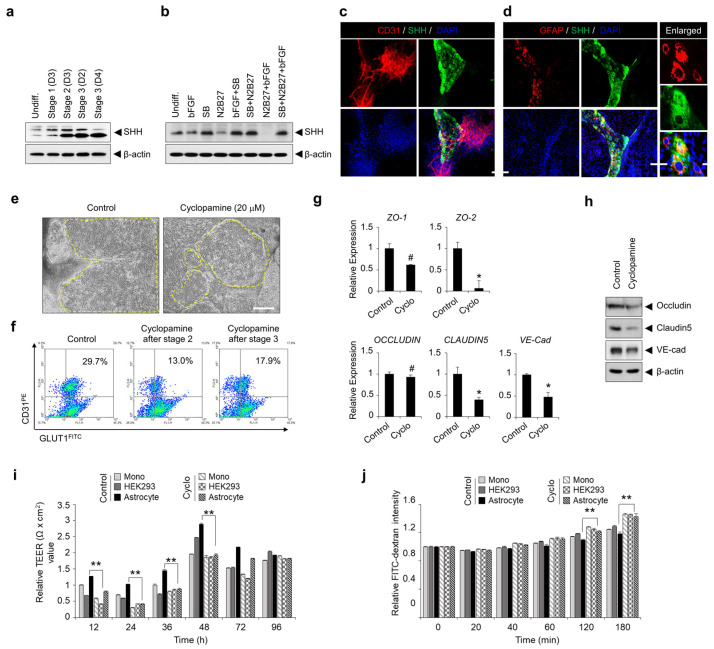
Sonic hedgehog is important for BBB function and is regulated through TGF-β signaling. (**a**,**b**) Sonic hedgehog (SHH) expression was determined by western blot analysis during differentiation (**a**) and according to several components (**b**). β-Actin was used as the loading control. Undiff, undifferentiated hPSCs; bFGF, 20 ng/mL bFGF; SB, 10 µM SB431542; N2B27, 1X N2B27 supplement. (**c**,**d**) Representative images during differentiation into BME-like cells from hPSCs stained with anti-CD31 (red) and anti-SHH (green) (**c**), and anti-GFAP (red) and anti-SHH (green) (**d**) at stage 3 are shown. Scale bar = 200 µm. (**e**) Morphological changes of differentiating cells treated with 20 µM cyclopamine were observed compared with the control under bright-field microscopy. Yellow circles indicate flattened cobblestone EC morphology. Scale bar = 200 µm. (**f**) The expression of CD31 and GLUT1 of differentiating cells treated with 20 µM cyclopamine was assessed by FACS analysis compared with control. (**g**,**h**) Expression of TJ molecules (ZO-1, ZO-2, OCCLUDIN, CLAUDIN5, and VE-cad) in hPSC-derived BME-like cells treated with or without 20 µM cyclopamine during differentiation by RT-PCR (**g**) and western blot analysis (**h**). Statistical significance is denoted by # *p* < 0.1, * *p* < 0.05 vs. control. Data are represented as mean ± SEM. (**i**) TEER measured on hPSC-derived BME-like cells treated with or without 20 µM cyclopamine when co-cultured with astrocytes or HEK293 cells under indirect-contact co-culture using transwell. Triplicate filters were used to calculate TEER values. Statistical significance is denoted by ** *p* < 0.01 vs. monoculture. Data are represented as mean ± SEM. (**j**) Permeability of FITC-dextran of hPSC-derived BME-like cells treated with or without 20 µM cyclopamine when co-cultured with astrocytes or HEK293 cells. Cyclo: cyclopamine. Statistical significance is denoted by ** *p* < 0.01 vs. astrocyte of control group. Data are represented as mean ± SEM.

To identify expression of SHH regulated by TGF-β signaling effects on differentiation into BME-like cells and BBB properties, we verified the SHH expression at each stage during differentiation. Interestingly, we observed that the expression of SHH was augmented after stage 2, which resulted in us wondering whether SHH is regulated by TGF-β signaling. To demonstrate this issue, hPSCs were cultured and differentiated in multiple conditions, and then we identified the expression pattern of SHH at final stage 3. Notably, although SHH is slightly expressed under basal condition, inhibition of TGF-β signaling by treatment of SB431542 significantly increased expression of SHH compared with the untreated group (Figure 5a,b). Moreover, we observed that SHH was mainly expressed on GFAP-expressing cells in the non-EC region (Figure 5c,d). We next determined whether TGF-β signaling inhibition-mediated SHH is involved in hPSC differentiation into BME-like cells and the acquisition of BBB properties. Treatment with cyclopamine, a blocker of SHH signaling, from stage 2 or stage 3, markedly decreased EC-like colonies and the CD31+GLUT1+ population as compared to the control (Figure 5e,f and Figure S4a,b). In addition, inhibition of SHH signaling reduced the expression of cell-to-cell junction genes, including ZO-1, ZO-2, OCCLUDIN, CLAUDIN5, and VE-cad (Figure 5g,h). Moreover, TEER value of BME-like cells from the differentiation condition with cyclopamine decreased despite of co-culture with astrocyte, and also significantly increased dextran permeability (Figure 5i,j). Taken together, these results demonstrate that SHH signaling might be critical for enhancing hPSC differentiation into functional BME-like cells and for the acquisition of BMEC properties.

## 4. Discussion

Although BMECs could be obtained from animal tissue and human biopsy, such cells have several hurdles for application to the BBB model. Animal sources have difficulty in representing the human BBB properties due to inevitable species differences [11,37]. Furthermore, human primary BMECs or immortalized human cell lines represent low barrier properties, such as TEER and tight junctions, unsuitable phenotype, and poor longevity [13,38]. Here, we suggest a novel and simple approach for the differentiation of hPSCs into ECs having definitive BMEC properties to establish and use BBB models. In addition, we draw attention to the finding that the cross-talk of TGF-β and SHH signaling during the co-differentiation of neural cells and ECs from hPSCs generates more reliable and reproducible BME-like cells. The well-established BME-like cells developed in our system showed long-term maintenance, proliferation, high functionality, and characteristics similar to those of pBMECs.

hPSCs can facilitate the efficient development of functional BME-like cells, which are promising for studying BBB development, physiology, neurological disease, and related drug discovery. However, until now, obtaining mature BME-like cells with desirable properties from hPSCs for clinical applications and for establishing BBB models has been challenging. To address these concerns, BME-like cells produced from hPSCs have been co-cultured with pericytes, astrocytes, or differentiating neural cells in the past [5,35,39,40]. However, the acquisition of functional BBB EC properties has remained unattainable. In this study, we developed a new differentiation protocol for the generation of mature BBB ECs using a co-differentiation system comprising both neural cells and ECs in combination with signal pathway regulators of cell–cell interaction, thus imitating the development environment of brain ECs. These cells are constantly communicating with each other and are influenced by interactions with soluble factors secreted by neighboring neural cells, which can maintain BBB homeostasis and integrity [41,42,43,44]. Astrocytes and neurons as NVUs play an important role in the maintenance of BBB function [1], and various signaling pathways, including Wnt, TGF-β, and SHH, are involved in BBB EC development, as well as BBB maturation [42]. Considering these points, we first induced mesodermal specification by treatment with the GSK-3β inhibitor BIO, as well as BMP4, Activin A, and 1X N2B27 components, at differentiation stage 1 to maintain Wnt-mediated β-catenin signaling activation, which is important for CNS vascular development [15,28,29,45], while suppressing differentiation into an ectoderm lineage. BIO treatment significantly upregulated the expression of mesoderm markers but downregulated the expression of ectoderm markers (Figure S1b). Then, the TGF-β signal inhibitor SB431542 was added along with basic fibroblast growth factor (bFGF) to induce co-differentiation into mesoderm and ectoderm lineages at differentiation stage 2. Interestingly, the inhibition of the TGF-β signaling pathway increased differentiation into ECs instead, as well as differentiation into mature neural cells (Figure 4). During BBB development, the TGF-β signaling pathway is essential for BBB EC maturation by inducing extracellular matrix expression in pericytes and promoting pericyte adhesion in ECs [42]. Among these, pericytes have a critical role for regulation of vessel stability, and BBB integrity and function [46]. Especially, downregulation of TGF-β signaling in ECs interacted with pericytes leads to BBB breakdown, following increase in BBB permeability and hemorrhage [47]. Furthermore, pericyte regulates the expression of LAMs, which diminish vascular permeability and infiltration of immune cells [48]. Inhibition of TGF-β signaling with SB431542 or soluble TGFβRII from hPSCs is required for differentiation into ECs and its vascular commitment [49]. However, we found that the inhibition of TGF signaling during PSC differentiation into ECs and neural cell co-differentiation is important for improving differentiation efficiency.

This study further provides insights into the mechanism by which the inhibition of TGF-β signaling regulates hPSC differentiation into BME-like cells and maintains BBB properties. The SHH pathway has been identified as important for BBB maturation, embryonic morphogenesis, neuronal guidance, and angiogenesis [42,50]. Previous studies have shown that SHH produced by astrocytes promotes BBB integrity by regulating the expression of TJ proteins such as occludin and claudin5 [25]. In our system, we also observed that SHH expression was prominently increased from differentiation stage 2 (Figure 5a) and was specifically induced on GFAP-expressing cells by the inhibition of the TGF-β signaling pathway (Figure 5d). Interestingly, inhibition of the SHH signaling pathway with cyclopamine resulted in a significant reduction in the differentiation efficiency of hPSCs into BME-like cells (Figure 5f and Figure S4). Moreover, the expression levels of TJ molecules such as ZO-1, ZO-2, OCCLUDIN, CLAUDIN5, and VE-cad decreased, which led to a significant decrease of TEER value in BME-like cells treated with cyclopamine compared with the untreated group; furthermore, the permeability of FITC-dextran was increased in BME-like cells differentiated with cyclopamine compared with the untreated group (Figure 5g–j). These results indicate that inhibition of the TGF-β signaling pathway in our differentiation system not only increases the differentiation efficiency of hPSCs into BME-like cells by promoting co-differentiation into ECs and neural cells, but also plays an important role in the maturation of the BBB by inducing SHH expression from astrocytes.

Our data also show that our BME-like cells have high functionality, long-term maintenance, and BBB properties similar to those of pBMECs. Although differences in technical aspects during differentiation using the same method cannot be ruled out, the BME-like cells from our system were similar to pBMECs. In detail, as shown in Figure S5, BME-like cells differentiated according to our system represented typically endothelial morphological phenotype, and formed a mature capillary-like tube structure compared with published protocol [5] (Figure S5a,b). Furthermore, BME-like cells showed higher BBB phenotype, such as BBB-related receptor, transporter, and tight junction molecules, compared to published protocol [5] (Figure S5c–e). In addition, we confirmed high similarity with pBMECs in gene expression patterns by RNA-sequence analysis. In particular, the expression levels of BBB EC transporters, membrane-specific proteins, and channel-specific markers in our BME-like cells were comparable to pBMECs. Furthermore, we confirmed that various transporters such as P-gp, BCRP, and MRP were active in these cells (Figure 3c,d). Meanwhile, since investigating the influence by fluid shear stress is one of the criteria for validating the barrier integrity of the BBB model, it would be further necessary to assess the barrier function of BME-like cells co-cultured with astrocytes in the presence of shear stress.

To our knowledge, this study is also the first to show that BME-like cells differentiated from hPSCs have immune quiescence, which is one of the important characteristics of BBB ECs. BBB ECs have a low response to inflammatory cytokines compared to non-CNS ECs, resulting in low induction of adhesion molecules, including ICAM1 and VCAM1, as well as low leukocyte adhesion. BME-like cells that differentiated from hPSCs in our system exhibited lower expression of ICAM1 and VCAM1 due to decreased NF-κB activation in response to inflammatory cytokines, resulting in decreased adhesion of monocytes (Figure 2e,f and Figure S2j). These results suggest that our BME-like cells have very similar characteristics to BBB ECs in terms of function and gene expression, reinforcing their utility in BBB model application.

In summary, this study presents a new, efficient approach for the production of BME-like cells from hPSCs. Our study also highlights the importance of cross-talk between neural population and ECs by TGF-β signaling inhibition-mediated SHH signaling activation. This cellular platform is useful for the establishment of in vitro BBB models and may also help boost studies on the functions of the BBB and the development of pharmaceuticals for applications in neurological disorders in future.

## Data Availability

The raw/processed data required to reproduce these findings are available from the corresponding author, Jeong-Ki Min (jekmin74@gmail.com).

## Supplementary Materials

The following supporting information can be downloaded at: https://www.mdpi.com/article/10.3390/bioengineering10101132/s1, Figure S1: Gene expression of lineage specific markers during differentiation and importance of GSK3-β inhibition to induce mesoderm lineage; Figure S2: Similarity of BMEC phenotype between BME-like cell and pBMECs; Figure S3: Differential expression of CD31 and GLUT1 with or without SB431542; Figure S4: Inhibition of SHH signaling pathway decreases differentiation potential into BME-like cells from hPSCs; Figure S5: Comparison of BMEC phenotype between BME-like cell and another protocol-derived brain EC; Table S1: List of antibodies used in this study; Table S2: List of primers used in this study.

## References

[1] Abbott N.J., Ronnback L., Hansson E. (2006). Astrocyte-endothelial interactions at the blood-brain barrier. Nat. Rev. Neurosci..

[2] Zhao Z., Nelson A.R., Betsholtz C., Zlokovic B.V. (2015). Establishment and Dysfunction of the Blood-Brain Barrier. Cell.

[3] Deeken J.F., Loscher W. (2007). The blood-brain barrier and cancer: Transporters, treatment, and Trojan horses. Clin. Cancer Res..

[4] Chow B.W., Gu C. (2015). The molecular constituents of the blood-brain barrier. Trends Neurosci..

[5] Lippmann E.S., Al-Ahmad A., Azarin S.M., Palecek S.P., Shusta E.V. (2014). A retinoic acid-enhanced, multicellular human blood-brain barrier model derived from stem cell sources. Sci. Rep..

[6] Sweeney M.D., Sagare A.P., Zlokovic B.V. (2018). Blood-brain barrier breakdown in Alzheimer disease and other neurodegenerative disorders. Nat. Rev. Neurol..

[7] Upadhyay R.K. (2014). Drug delivery systems, CNS protection, and the blood brain barrier. BioMed Res. Int..

[8] Bagchi S., Chhibber T., Lahooti B., Verma A., Borse V., Jayant R.D. (2019). In-vitro blood-brain barrier models for drug screening and permeation studies: An overview. Drug Des. Dev. Ther..

[9] Wilhelm I., Krizbai I.A. (2014). In vitro models of the blood-brain barrier for the study of drug delivery to the brain. Mol. Pharm..

[10] Warren M.S., Zerangue N., Woodford K., Roberts L.M., Tate E.H., Feng B., Li C., Feuerstein T.J., Gibbs J., Smith B. (2009). Comparative gene expression profiles of ABC transporters in brain microvessel endothelial cells and brain in five species including human. Pharmacol. Res..

[11] Helms H.C., Abbott N.J., Burek M., Cecchelli R., Couraud P.O., Deli M.A., Forster C., Galla H.J., Romero I.A., Shusta E.V. (2016). In vitro models of the blood-brain barrier: An overview of commonly used brain endothelial cell culture models and guidelines for their use. J. Cereb. Blood Flow Metab..

[12] Di Marco A., Vignone D., Gonzalez Paz O., Fini I., Battista M.R., Cellucci A., Bracacel E., Auciello G., Veneziano M., Khetarpal V. (2020). Establishment of an in Vitro Human Blood-Brain Barrier Model Derived from Induced Pluripotent Stem Cells and Comparison to a Porcine Cell-Based System. Cells.

[13] Cecchelli R., Berezowski V., Lundquist S., Culot M., Renftel M., Dehouck M.P., Fenart L. (2007). Modelling of the blood-brain barrier in drug discovery and development. Nat. Rev. Drug Discov..

[14] Qian T., Maguire S.E., Canfield S.G., Bao X., Olson W.R., Shusta E.V., Palecek S.P. (2017). Directed differentiation of human pluripotent stem cells to blood-brain barrier endothelial cells. Sci. Adv..

[15] Lippmann E.S., Azarin S.M., Kay J.E., Nessler R.A., Wilson H.K., Al-Ahmad A., Palecek S.P., Shusta E.V. (2012). Derivation of blood-brain barrier endothelial cells from human pluripotent stem cells. Nat. Biotechnol..

[16] Aday S., Cecchelli R., Hallier-Vanuxeem D., Dehouck M.P., Ferreira L. (2016). Stem Cell-Based Human Blood-Brain Barrier Models for Drug Discovery and Delivery. Trends Biotechnol..

[17] Lippmann E.S., Al-Ahmad A., Palecek S.P., Shusta E.V. (2013). Modeling the blood-brain barrier using stem cell sources. Fluids Barriers CNS.

[18] Lauschke K., Frederiksen L., Hall V.J. (2017). Paving the Way Toward Complex Blood-Brain Barrier Models Using Pluripotent Stem Cells. Stem Cells Dev..

[19] Eichmann A., Thomas J.L. (2013). Molecular parallels between neural and vascular development. Cold Spring Harb. Perspect. Med..

[20] Paredes I., Himmels P., Ruiz de Almodovar C. (2018). Neurovascular Communication during CNS Development. Dev. Cell.

[21] Park K.S. (2011). Tgf-Beta family signaling in embryonic stem cells. Int. J. Stem Cells.

[22] Smith J.R., Vallier L., Lupo G., Alexander M., Harris W.A., Pedersen R.A. (2008). Inhibition of Activin/Nodal signaling promotes specification of human embryonic stem cells into neuroectoderm. Dev. Biol..

[23] Chambers S.M., Fasano C.A., Papapetrou E.P., Tomishima M., Sadelain M., Studer L. (2009). Highly efficient neural conversion of human ES and iPS cells by dual inhibition of SMAD signaling. Nat. Biotechnol..

[24] Zhong Q., Laco F., Liao M.C., Woo T.L., Oh S.K.W., Chai C.L.L. (2018). Influencing the Fate of Cardiac and Neural Stem Cell Differentiation Using Small Molecule Inhibitors of ALK5. Stem Cells Transl. Med..

[25] Alvarez J.I., Dodelet-Devillers A., Kebir H., Ifergan I., Fabre P.J., Terouz S., Sabbagh M., Wosik K., Bourbonniere L., Bernard M. (2011). The Hedgehog pathway promotes blood-brain barrier integrity and CNS immune quiescence. Science.

[26] Lim M.H., Jeung I.C., Jeong J., Yoon S.J., Lee S.H., Park J., Kang Y.S., Lee H., Park Y.J., Lee H.G. (2016). Graphene oxide induces apoptotic cell death in endothelial cells by activating autophagy via calcium-dependent phosphorylation of c-Jun N-terminal kinases. Acta Biomater..

[27] Aubert J., Dunstan H., Chambers I., Smith A. (2002). Functional gene screening in embryonic stem cells implicates Wnt antagonism in neural differentiation. Nat. Biotechnol..

[28] Liebner S., Corada M., Bangsow T., Babbage J., Taddei A., Czupalla C.J., Reis M., Felici A., Wolburg H., Fruttiger M. (2008). Wnt/beta-catenin signaling controls development of the blood-brain barrier. J. Cell Biol..

[29] Stenman J.M., Rajagopal J., Carroll T.J., Ishibashi M., McMahon J., McMahon A.P. (2008). Canonical Wnt signaling regulates organ-specific assembly and differentiation of CNS vasculature. Science.

[30] Davidson K.C., Adams A.M., Goodson J.M., McDonald C.E., Potter J.C., Berndt J.D., Biechele T.L., Taylor R.J., Moon R.T. (2012). Wnt/beta-catenin signaling promotes differentiation, not self-renewal, of human embryonic stem cells and is repressed by Oct4. Proc. Natl. Acad. Sci. USA.

[31] Lian X., Bao X., Al-Ahmad A., Liu J., Wu Y., Dong W., Dunn K.K., Shusta E.V., Palecek S.P. (2014). Efficient differentiation of human pluripotent stem cells to endothelial progenitors via small-molecule activation of WNT signaling. Stem Cell Rep..

[32] Abdul Muneer P.M., Alikunju S., Szlachetka A.M., Murrin L.C., Haorah J. (2011). Impairment of brain endothelial glucose transporter by methamphetamine causes blood-brain barrier dysfunction. Mol. Neurodegener..

[33] Stebbins M.J., Wilson H.K., Canfield S.G., Qian T., Palecek S.P., Shusta E.V. (2016). Differentiation and characterization of human pluripotent stem cell-derived brain microvascular endothelial cells. Methods.

[34] Daneman R., Prat A. (2015). The blood-brain barrier. Cold Spring Harb. Perspect. Biol..

[35] Lippmann E.S., Weidenfeller C., Svendsen C.N., Shusta E.V. (2011). Blood-brain barrier modeling with co-cultured neural progenitor cell-derived astrocytes and neurons. J. Neurochem..

[36] Di Marco A., Gonzalez Paz O., Fini I., Vignone D., Cellucci A., Battista M.R., Auciello G., Orsatti L., Zini M., Monteagudo E. (2019). Application of an in Vitro Blood-Brain Barrier Model in the Selection of Experimental Drug Candidates for the Treatment of Huntington’s Disease. Mol. Pharm..

[37] Syvanen S., Lindhe O., Palner M., Kornum B.R., Rahman O., Langstrom B., Knudsen G.M., Hammarlund-Udenaes M. (2009). Species differences in blood-brain barrier transport of three positron emission tomography radioligands with emphasis on P-glycoprotein transport. Drug Metab. Dispos..

[38] Weksler B.B., Subileau E.A., Perriere N., Charneau P., Holloway K., Leveque M., Tricoire-Leignel H., Nicotra A., Bourdoulous S., Turowski P. (2005). Blood-brain barrier-specific properties of a human adult brain endothelial cell line. FASEB J..

[39] Nakagawa S., Deli M.A., Kawaguchi H., Shimizudani T., Shimono T., Kittel A., Tanaka K., Niwa M. (2009). A new blood-brain barrier model using primary rat brain endothelial cells, pericytes and astrocytes. Neurochem. Int..

[40] Yamamizu K., Iwasaki M., Takakubo H., Sakamoto T., Ikuno T., Miyoshi M., Kondo T., Nakao Y., Nakagawa M., Inoue H. (2017). In Vitro Modeling of Blood-Brain Barrier with Human iPSC-Derived Endothelial Cells, Pericytes, Neurons, and Astrocytes via Notch Signaling. Stem Cell Rep..

[41] Ronaldson P.T., Davis T.P. (2012). Blood-brain barrier integrity and glial support: Mechanisms that can be targeted for novel therapeutic approaches in stroke. Curr. Pharm. Des..

[42] Obermeier B., Daneman R., Ransohoff R.M. (2013). Development, maintenance and disruption of the blood-brain barrier. Nat. Med..

[43] Blanchette M., Daneman R. (2015). Formation and maintenance of the BBB. Mech. Dev..

[44] Geranmayeh M.H., Rahbarghazi R., Farhoudi M. (2019). Targeting pericytes for neurovascular regeneration. Cell Commun. Signal..

[45] Daneman R., Agalliu D., Zhou L., Kuhnert F., Kuo C.J., Barres B.A. (2009). Wnt/beta-catenin signaling is required for CNS, but not non-CNS, angiogenesis. Proc. Natl. Acad. Sci. USA.

[46] Winkler E.A., Bell R.D., Zlokovic B.V. (2011). Central nervous system pericytes in health and disease. Nat. Neurosci..

[47] Li F., Lan Y., Wang Y., Wang J., Yang G., Meng F., Han H., Meng A., Wang Y., Yang X. (2011). Endothelial Smad4 maintains cerebrovascular integrity by activating N-cadherin through cooperation with Notch. Dev. Cell.

[48] Daneman R., Zhou L., Kebede A.A., Barres B.A. (2010). Pericytes are required for blood-brain barrier integrity during embryogenesis. Nature.

[49] James D., Nam H.S., Seandel M., Nolan D., Janovitz T., Tomishima M., Studer L., Lee G., Lyden D., Benezra R. (2010). Expansion and maintenance of human embryonic stem cell-derived endothelial cells by TGFbeta inhibition is Id1 dependent. Nat. Biotechnol..

[50] Fuccillo M., Joyner A.L., Fishell G. (2006). Morphogen to mitogen: The multiple roles of hedgehog signalling in vertebrate neural development. Nat. Rev. Neurosci..

