# Supplementary material for: TGF-β and SHH Regulate Pluripotent Stem Cell Differentiation into Brain Microvascular Endothelial Cells in Generating an In Vitro Blood–Brain Barrier Model
Source: Bioengineering (Basel). 2023 Sep 27;10(10):1132. doi: 10.3390/bioengineering10101132 (PMC10604460; doi:10.3390/bioengineering10101132)
Supplement: Supplementary file 1 [file bioengineering-10-01132-s001.zip › bioengineering-2584362-supplementary.pdf]

Supplementary Figures

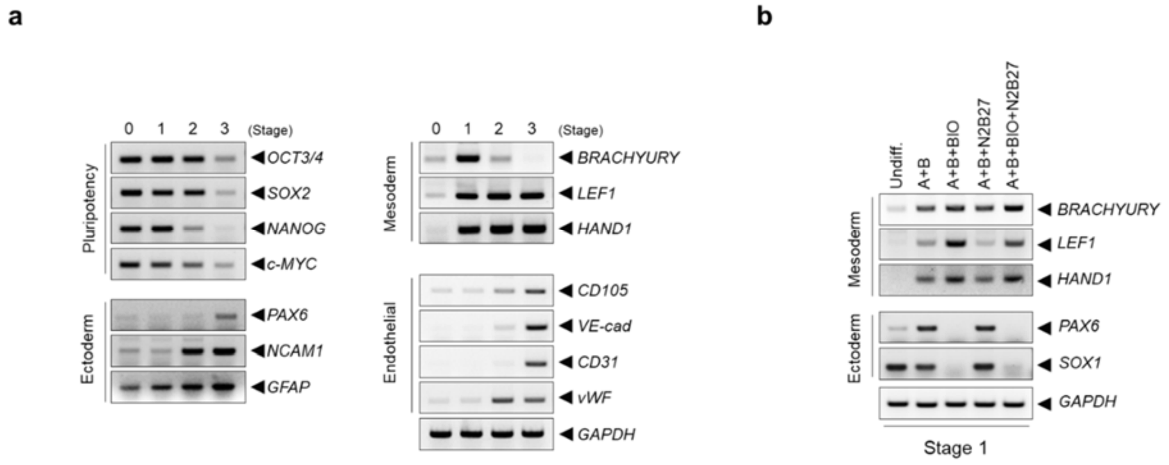

Scheme 1. Gene expression of lineage specific markers during differentiation and importance of GSK3-β inhibition to induce mesoderm lineage. a Gene expression of pluripotent stem cell-, ectoderm-, mesoderm-, and endothelial-related markers at differentiation stages by RT-PCR. b Expression of mesoderm and ectoderm markers after treatment with the GSK3-β inhibitor BIO by RT-PCR. A: Activin A, B: BMP4.

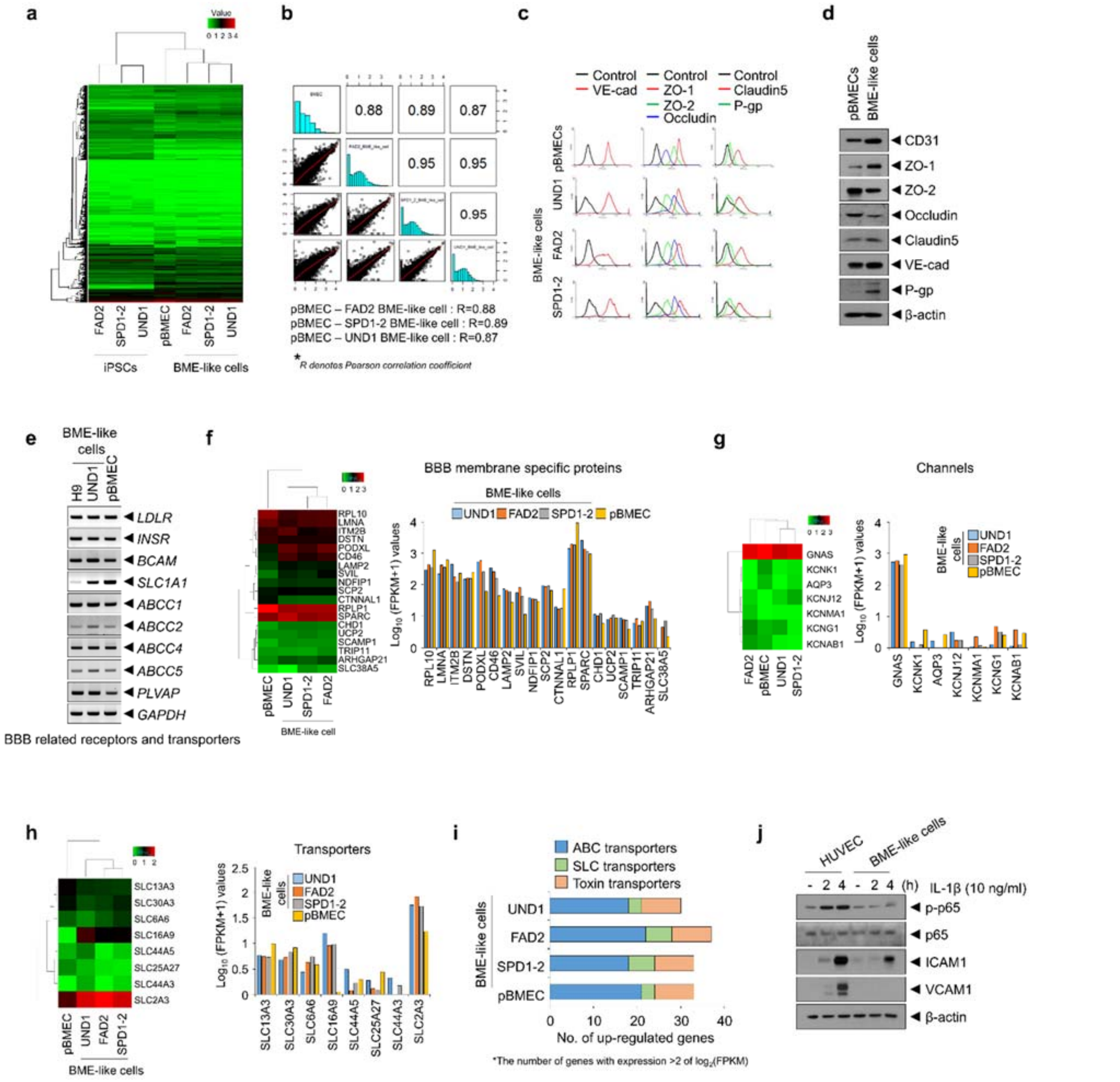

Scheme 2. Similarity of BMEC phenotype between BME-like cell and pBMEC. a, **b** Heatmaps of whole-gene expression in hPSCs, pBMECs, and hPSC-derived BME-like cells by RNA-sequence analysis. Red indicates the highest level of similarity between samples, and green indicates the lowest level of similarity (**a**). Scatter plots showing correlation ratios between pBMECs and hPSC-derived BME-like cells (**b**). **c**, **d** Expression of tight junction markers in hPSC-derived BME-like cells compared with pBMECs by FACS (**c**) and western blot analysis (**d**). **e** The gene expression of BBB-related receptors and transporters in pBMECs and hPSC-derived BME-like cells was determined by RT-PCR. **f–h** Expression values (FPKM) of genes encoding BBB membrane-specific proteins (**f**), channels (**g**), and transporters (**h**) in pBMECs and hPSC-derived BME-like cells were $\log_{10}$ transformed and then represented by a heatmap graph. **i** Comparison of the gene expression of ABC transporters, SLC transporters, and toxin transporters among pBMECs and hPSC-derived BME-like cells. The graph represents the number of genes with expression > 2-fold of $\log_2$ (FPKM). **j** Expression of p-p65, ICAM1, and VCAM1 in HUVECs and hPSC-derived BME-like cells treated with or without 10 ng/mL IL-1β for the indicated times. Expression levels were identified by western blot analysis. β-Actin was used as the loading control.

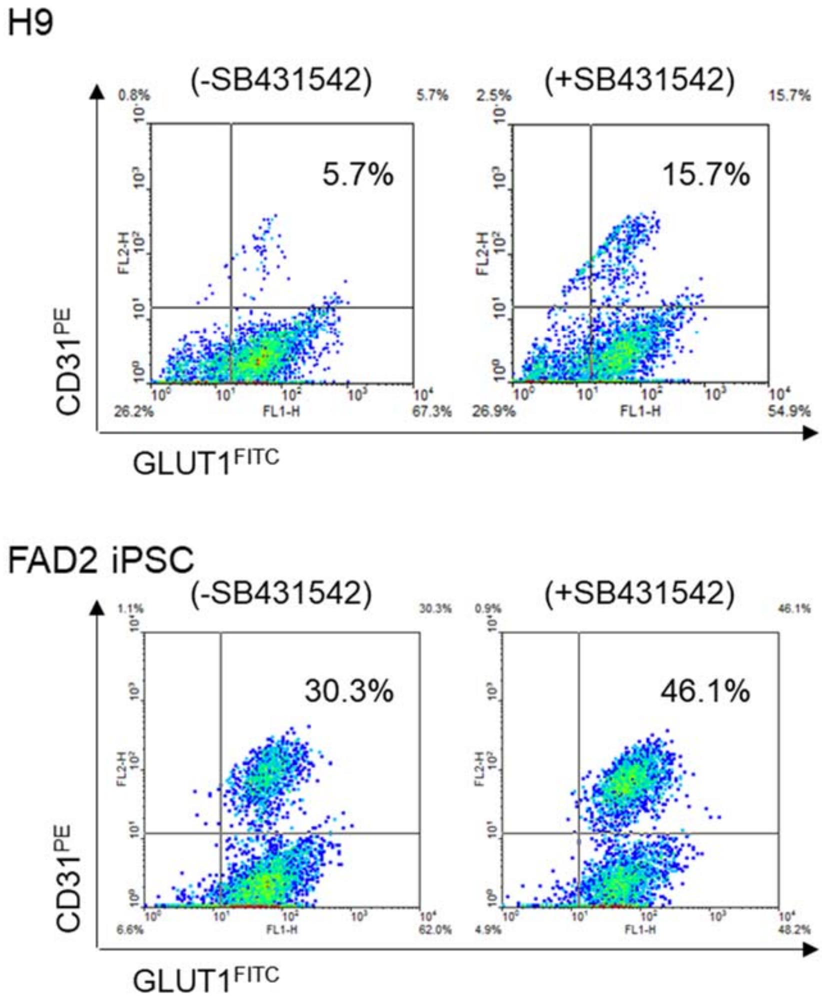

Scheme 3. CD31 and GLUT1 expression in differentiating cells into BME-like cells from H9 or FAD iPSCs treated with or without 10 μM SB431542 was assessed by FACS analysis.

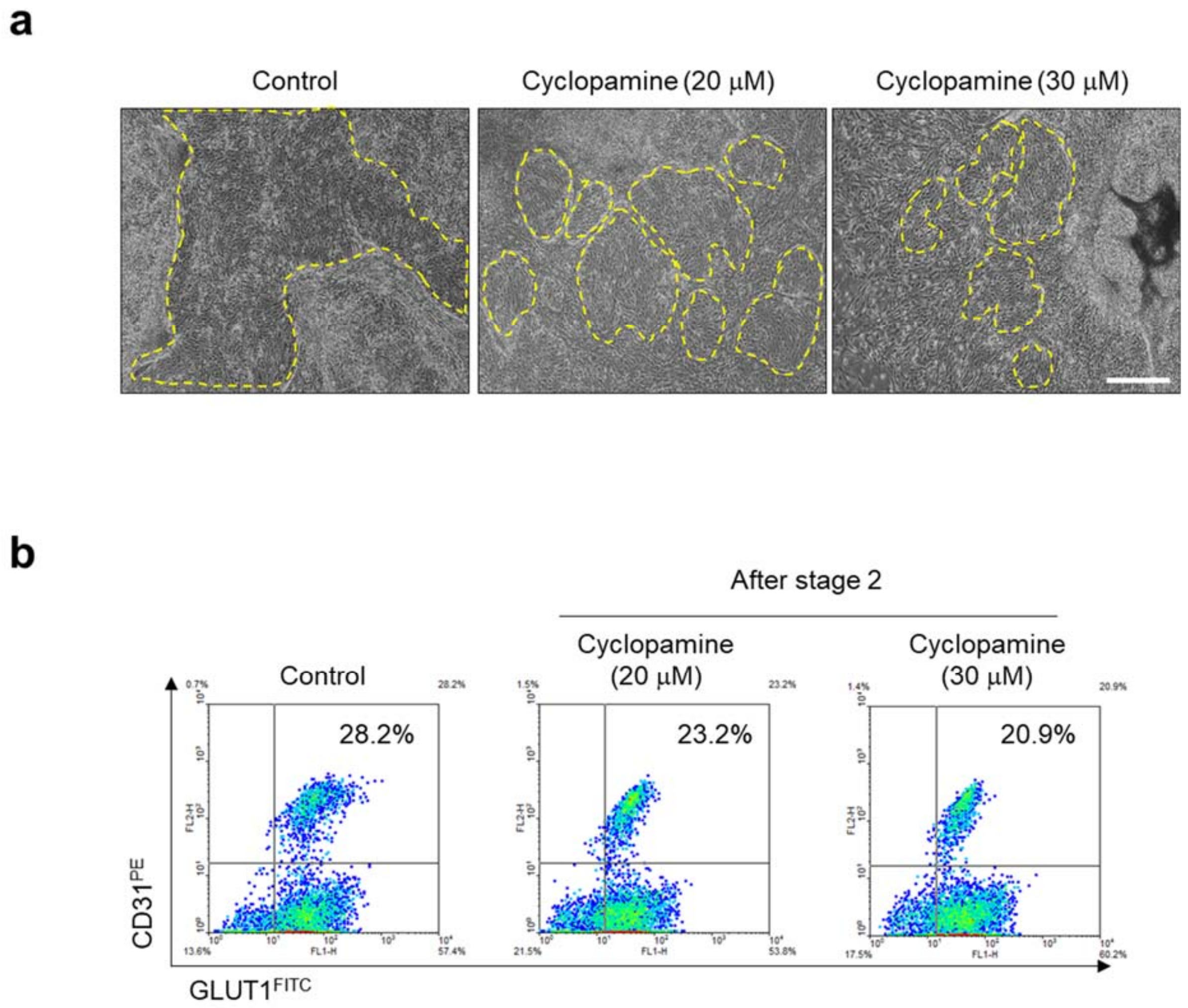

Scheme 4. Inhibition of SHH signaling pathway decrease differentiation potential into BME-like cells from hPSCs. a Morphological changes of differentiating cells into BME-like cells from hPSCs treated with cyclopamine (20 μM or 30 μM) were observed compared with the control under bright-field microscopy. **b** CD31 and GLUT1 expression in differentiating cells into BME-like cells from hPSCs treated with or without cyclopamine (20 μM or 30 μM) after stage 2 was assessed by FACS analysis compared with the control.

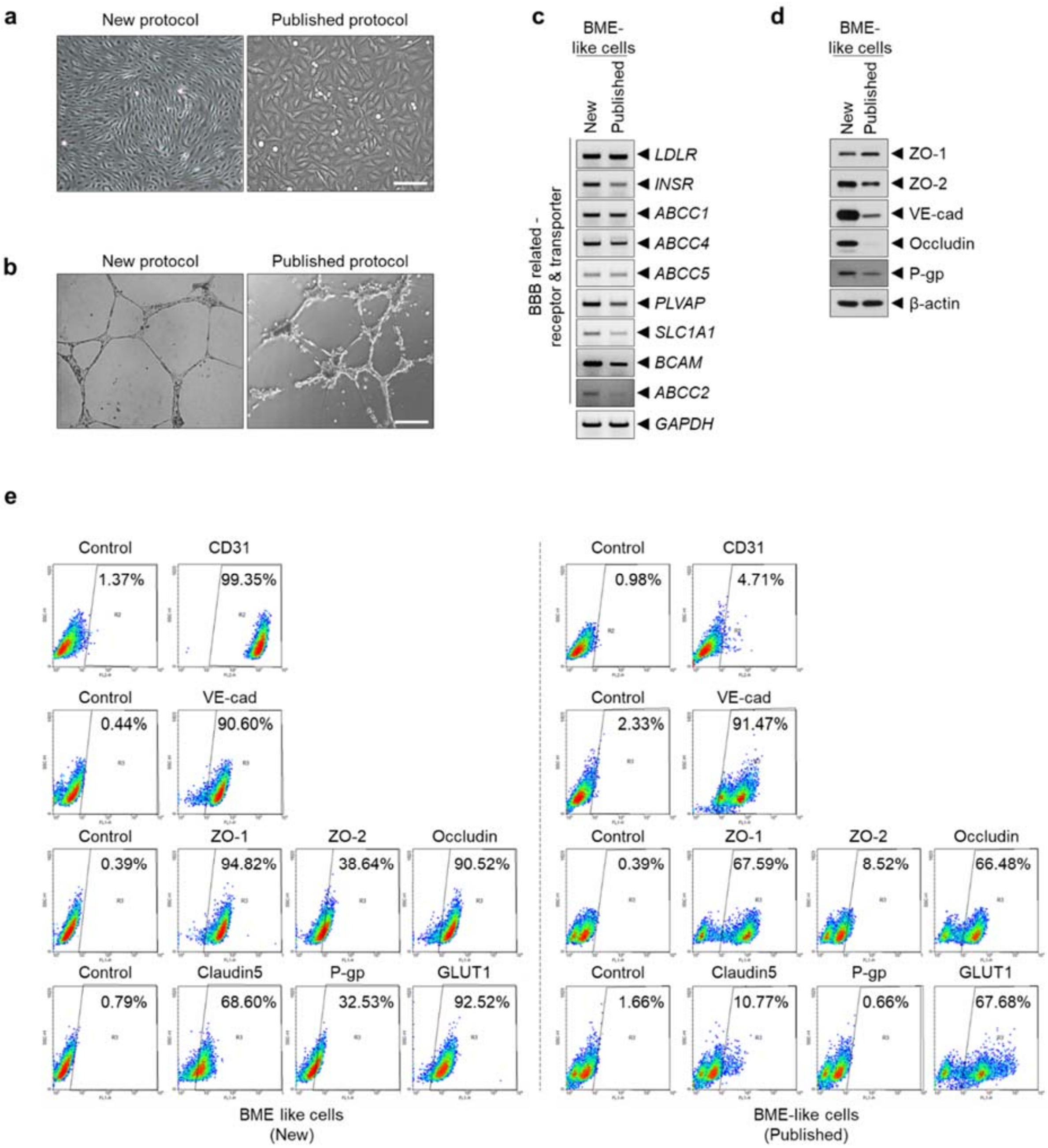

Scheme 5. Comparison of BMEC phenotype between BME-like cell and another protocol-derived brain EC. a–e Comparison of brain endothelial-like cells using new and published protocol[1]. Morphology (**a**), capillary-like tube formation (**b**), gene expression of receptors and transporters by RT-PCR (**c**), expression of tight junction proteins by western blot analysis (**d**), and BMEC-related marker expression by FACS analysis (**e**).

Scheme 1. List of antibodies used in this study.

Antibodies	Company	Dilution and application	Catalog No.
PECAM-1	Santa Cruz	1:50 for ICC	Cat#SC-133091
		1:100 for WB	
PE Mouse Anti-Human CD31	BD Bioscience	1:100 for FACS	Cat#555446
VE-cadherin	Santa Cruz	1:50 for ICC	Cat#SC-6458
		1:100 for FACS	
		1:100 for WB	

ZO-1	Thermo	1:100 for ICC and FACS<br>1:1000 for WB	Cat#40-2200
ZO-2	Thermo	1:50 for ICC and FACS<br>1:1000 for WB	Cat#71-1400
Occludin	Thermo	1:100 for ICC and FACS<br>1:1000 for WB	Cat#71-1500
Claudin-5	Thermo	1:50 for ICC and FACS<br>1:1000 for WB	Cat#35-2500
Anti-Glucose Transporter GLUT1 antibody	Abcam	1:100 for ICC and FACS<br>1:1000 for WB	Cat#ab40084
Anti-P-Glycoprotein Mouse mAb (C219)	Millipore	1:100 for ICC and FACS<br>1:1000 for WB	Cat#517310
Anti-β-Tubulin III antibody	Sigma	1:500 for ICC	Cat#T2200
Anti-Nestin Antibody, clone 10C2	Millipore	1:500 for ICC	Cat#MAB5326
Anti-Nestin-PE Antibody, clone 10C2	Millipore	1:100 for FACS	Cat#FCMAB313PE
Monoclonal Anti-Glial Fibrillary Acidic Protein (GFAP) antibody	Sigma	1:200 for ICC	Cat#G3893
Anti-Sonic Hedgehog Antibody	Millipore	1:100 for ICC<br>1:1000 for WB	Cat#04-971
Recombinant Anti-Sonic Hedgehog antibody	Abcam	1:100 for ICC	Cat#Ab53281
p-NFκB (p-p65)	Cell Signaling Technology	1:1000 for WB	Cat#3033S
NFκB (p65)	Cell Signaling Technology	1:1000 for WB	Cat#8242
ICAM-1	Santa Cruz	1:1000 for WB	Cat#SC-7981
VCAM-1	Santa Cruz	1:1000 for WB	Cat#SC-8304
Human E-cadherin	BD Bioscience	1:1000 for WB	Cat#180215
Claudin-4	Santa Cruz	1:1000 for WB	Cat#SC-376643
Claudin-6	Santa Cruz	1:1000 for WB	Cat#SC-393671
Beta Actin (C-term) Antibody	AbClon	1:5000 for WB	Cat#abc-2004

Scheme 2. List of primers used in this study.

Gene		Sequence (5' ->3')
LDLR	Forward	GGCAGAGGAAATGAGAAGAA
	Reverse	CATCTGTCTCGAGGGGTAG
INSR	Forward	TCTGATCGTGCTGTATGAAG
	Reverse	CGGGACGTCTAAATAGTCTG
BCAM	Forward	GCTTTCCTTACCTCTAAACAG
	Reverse	GAAGGTGATAGAACTGAGCG
SLC1A1	Forward	GATACTCCAAGGTGGCATAG

	Reverse	TGATACCAGCTTATGTCGTG
ABCC1	Forward	CAGATGACACCTCTCAACAA
	Reverse	GATCCCTGAAGACTGAACTC
ABCC2	Forward	ATATAAGAAGGCATTGACCC
	Reverse	ATCTGTAGAACACTTGACCA
ABCC4	Forward	CTCACGCGTGTTCTTCTG
	Reverse	TGTTACTAAGACGAAGTGCC
ABCC5	Forward	CCGCTATAAAGGCTTGTTTTG
	Reverse	CCATGATGGTACTTTCCCTT
PLVAP	Forward	TCTTCATGGTCTATGGCAAC
	Reverse	TTGATCTCTGCATTGCTTCT
OCT3/4	Forward	TCGTGCAGGCCCGAAAGAGA
	Reverse	TGGCGCCGGTTACAGAACCA
NANOG	Forward	ACCTGGAGCAACCAGACCCA
	Reverse	AGCTTCCAAGGCAGCCTCCA
SOX2	Forward	AAAAACAGCCCGGACCGCGT
	Reverse	TCCGCCGGGGCCGGTATTTA
c-MYC	Forward	CCCAGGTCCTCGGACACCGA
	Reverse	TGCTCCTCTGCTTGGACGGACA
BRACHYURY	Forward	AGCCACAATGCCAGCCCACC
	Reverse	GCCGCCCCTTCGTACAGTGG
LEF1	Forward	CGGACACGAGGTGGCCAGAC
	Reverse	ACCGCATGGGATGGCTGCAC
HAND1	Forward	TCCCTTTTCCGCTTGCTCTC
	Reverse	CATCGCCTACCTGATGGACG
PAX6	Forward	AGCGGGAGTGCCCGTCCATC
	Reverse	GGTTGCCCTGGCACCGAAGT
NCAM1	Forward	ACGGAGGAGGAGAGGACCCCA
	Reverse	CGTTCTCCTTTGTCTGTGTGGCG
GFAP	Forward	CCTCTCCCTGGCTCGAATG
	Reverse	GGAAGCGAACCTTCTCGATGTA
SOX1	Forward	CCACTCATTCGTTAAAGTGAT
	Reverse	CGGACTAAGTCGTAGTGGTGC
NESTIN	Forward	GCGGCTGCGGGCTACTGAAA
	Reverse	TCTTGGAGCCACCGCCAGGT
PDGFRβ	Forward	GAAGCTACATCTGCAAAACC
	Reverse	CAGCTCAGCAAATTGTAGTG
NEUROD1	Forward	GCAGCGCTGGAGCCCTTCTT
	Reverse	GATCCGTGGCTTTGGGCCCC
TUBB3	Forward	GGCCAAGTTCTGGGAAGTCA
	Reverse	CGAGTCGCCCACGTAGTTG
CD105	Forward	CCTTGATCCAGACAAAGTGT
	Reverse	GAGCTCGACAGGATATTGAC
CDH5	Forward	TGGAGAAGTGGCATCAGTCAACA
	Reverse	TCTACAATCCCTTGCAGTGTGAG
CD31	Forward	ACCCTGCAGTGCTTCGCGG
	Reverse	GTGTCACCCTGGGACTGGGC
vWF	Forward	CAAGGTACCTGAGAGAGGAG
	Reverse	GTCAATGGAGTAVATGGCTT
VE-cad	Forward	TGGAGAAGTGGCATCAGTCAACA

	Reverse	TCTACAATCCCTTGCAGTGTGAG
ZO-1	Forward	CTCCTGGATTTGGATTTGGA
	Reverse	TCAGGACGACTTACTGGTAT
ZO-2	Forward	CCAGATTCTGAAGGTGAACA
	Reverse	GTGGAGTTTCCTTCTCACAT
OCCLUDIN	Forward	GTGGTAACTTTGAGACACCT
	Reverse	CCAAACGGGAGAGTTCTTTA
CLAUDIN5	Forward	GACGTAGTTCTTCTTGTCGT
	Reverse	TGCTCTACCTGTTTTGCG
GAPDH	Forward	TGCCTCCTGCACCACCAA
	Reverse	ACACGTTGGCAGTGGGGA

Reference

5 Lippmann, E.S., Al-Ahmad, A., Azarin, S. M., Palecek, S. P. & Shusta, E. V. A retinoic acid-enhanced, multicellular human blood-brain barrier model derived from stem cell sources. *Sci. Rep.* **4**, 4160, http://doi:10.1038/srep04160 (2014).
